# Radionuclide Transport and Uptake in Coastal Aquatic Ecosystems: A Comparison of a 3D Dynamic Model and a Compartment Model

**DOI:** 10.1007/s13280-013-0398-2

**Published:** 2013-04-26

**Authors:** Anders Christian Erichsen, Lena Konovalenko, Flemming Møhlenberg, Rikke Margrethe Closter, Clare Bradshaw, Karin Aquilonius, Ulrik Kautsky

**Affiliations:** 1 Ecological and Environmental Department, DHI, Agern Allé 5, 2970 Hørsholm, Denmark; 2 The Department of Ecology, Environment and Plant Sciences, Stockholm University, 106 91 Stockholm, Sweden; 3 Swedish Nuclear Fuel and Waste Management Co. (SKB), Box 250, 101 24 Stockholm, Sweden; 4 Studsvik Nuclear AB, 611 82 Nyköping, Sweden

**Keywords:** Steady state, Biosphere, Process modeling, Bioaccumulation, Point source

## Abstract

In safety assessments of underground radioactive waste repositories, understanding radionuclide fate in ecosystems is necessary to determine the impacts of potential releases. Here, the reliability of two mechanistic models (the compartmental K-model and the 3D dynamic D-model) in describing the fate of radionuclides released into a Baltic Sea bay is tested. Both are based on ecosystem models that simulate the cycling of organic matter (carbon). Radionuclide transfer is linked to adsorption and flows of carbon in food chains. Accumulation of Th-230, Cs-135, and Ni-59 in biological compartments was comparable between the models and site measurements despite differences in temporal resolution, biological state variables, and partition coefficients. Both models provided confidence limits for their modeled concentration ratios, an improvement over models that only estimate means. The D-model enables estimates at high spatio-temporal resolution. The K-model, being coarser but faster, allows estimates centuries ahead. Future developments could integrate the two models to take advantage of their respective strengths.

**Electronic supplementary material:**

The online version of this article (doi:10.1007/s13280-013-0398-2) contains supplementary material, which is available to authorized users.

## Introduction

Nuclear power plants and nuclear waste storages facilities undergo frequent safety assessments to explore the fate of actual or hypothetical releases of radionuclides from the facilities. The aim of a safety analysis is to ensure that the risks to man (Avila et al. 2013) and the environment (Torudd and Saetre 2013) are negligible. A final repository for spent nuclear fuel is currently being planned at Forsmark, Sweden. For such a facility, this means that the time frame is up to a million years (Kautsky et al. 2013) and may include several periods of glaciation (Näslund et al. 2013) and long-term changes such as shore-line displacement and succession of the landscape from marine ecosystems, through lakes and mires, to terrestrial ecosystems (Lindborg et al. 2013), as well as changes in expected human behavior (Saetre et al. 2013). Usually, such facilities are located or planned close to the coast and will be close to the coast over most of the timeframe of their existence (Kautsky et al. 2013; Näslund et al. 2013). Thus, the fate of release of radionuclides to the biosphere and particularly the coastal marine ecosystem is of interest.

In marine ecosystems, radionuclides will disperse with currents, accumulate in biota, and be adsorbed by particles and sediment depending on local conditions and radionuclide properties. The major processes are water transport, dispersion due to diffusion and mixing, interaction of dissolved radionuclides with suspended matter and sediments, and transfer from the abiotic components to biota and between different components within biota (e.g., Bendoricchio and Jørgensen 2001; Jørgensen and Fath 2011).

For aquatic and terrestrial ecosystems, models constitute obvious tools to link observations and understanding of different processes and to predict future radionuclide distributions and concentrations (Monte et al. 2009). Depending on their scope, models can focus on: radionuclide transport with currents and exchange with sediments (Bulgakov et al. 2002; Håkanson and Monte 2003), assessment of radionuclide risks to organisms using a non-mechanistic approach to describe accumulation in various organisms based on distribution constants (e.g., concentration ratio, CR) between water and organisms (Lepicard et al. 2004; Brown et al. 2008; Heling and Bezhenar 2009; Avila et al. 2010, 2013), or on a mechanistic approach that takes into account ecological processes and transfers between organisms in the food web (Kryshev and Ryabov 2000; Koulikov and Meili 2003; Kumblad et al. 2006; Sandberg et al. 2007).

Non-mechanistic models are dependent on empirical estimates of partition coefficients of radionuclides to particles or organisms. The partition coefficients lump together radionuclide-specific processes with non-radionuclide-specific processes, which makes adaptation to other sites, spatial scales, and time frames difficult. Moreover, for many radionuclides it is difficult to obtain empirical partition coefficients, and inferences from other elements are necessary (Nordén et al. 2010; Avila et al. 2013). Mechanistic models, on the other hand, are less dependent on partition coefficients, and uptake can be scaled according to the ecosystem and other parameters like, e.g., water turnover. However, such models require a more detailed knowledge of the ecosystem and are usually structurally more complex (see Kumblad et al. 2006 for discussion).

In this article, we compare two different modeling approaches applied to the same area, namely a shallow coastal bay in the Baltic Proper near Forsmark, Sweden, where a geological final repository for high-level, long-lived radioactive waste (primarily, spent nuclear fuel) is planned at the coast (Kautsky et al. 2013). Both models simulate radionuclide distributions in the coastal ecosystem from a continuous point source release of 1 Bq y^−1^. They are mechanistic and driven by ecosystem models describing fluxes of carbon in the ecosystem, but the models differ in their spatio-temporal resolution and also in the number and type of biological state variables (organism groups) included.

Our aims are: (i) to evaluate the ability of the two models to estimate successfully radionuclide CRs in the coastal ecosystem and (ii) to identify similarities and differences, advantages, disadvantages, and complementarities of the two modeling approaches. Such information will be valuable when planning future impact assessments, in particular in addressing uncertainties of simple models versus complex ones.

## Materials and Methods

### Study Area

**Fig. 1:**
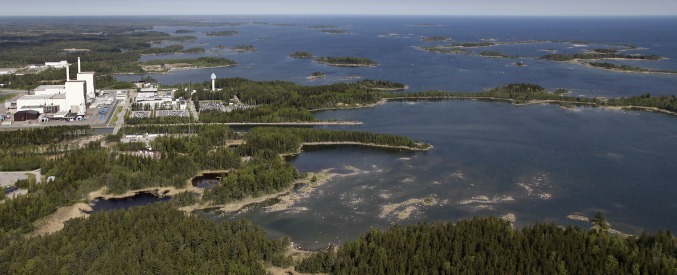
View of the Öregrundsgrepen in the Bothnian Sea. On the left side unit 1 of Forsmark nuclear power plant and in front the cooling channel inlet. A small archipelago extends to the open toward north. Photo by Lasse Modin

The coastal area considered in this study is a sub-basin of a shallow coastal bay in the Baltic Proper near Forsmark, Sweden. The area has a surface area of 11.5 km^2^ and is known as “basin 116,” one of 28 basins (“biosphere objects”) in the Forsmark area (Brydsten 2006; Lindborg et al. 2013) used in the safety assessment of a geological repository for spent nuclear fuel. Its location is shown in Kautsky et al. (2013, their Fig. 3). The photo in Fig. 1 provides a general view of the area. The marine ecosystems at the site and other important site data are described in Aquilonius (2010), who summarizes site data such as hydrodynamics, chemical and physical characteristics, biota types and biomass, as well as quantification of ecosystem processes. Elemental transfers in this area have also been studied (Bradshaw et al. 2012).

### Overview of the Two Models

The K-model is an ecological marine radionuclide transport model of a coastal food web developed by Kumblad et al. (2003, 2006), explained detail in Kumblad et al. (2003) and Kumblad and Kautsky (2004). For this study, the K-model has been improved and applied in the newer software package Ecolego 5 (Broed and Xu 2008). It is a food-web model that describes the biomass distribution and the carbon dynamics of the ecosystem, and includes both biotic and abiotic compartments. Radionuclides follow the flow of carbon in the ecosystem.

The 3D hydrodynamic MIKE3-FM model (Graham and Butts 2005; Butts and Graham 2008) and associated ecosystem model developed in ECOLab (D-model) were adapted and set-up to represent the specific water exchange and ecosystem conditions in Forsmark area. Resolution of the model varies from less than 20 m (near the coast) to more than 100 m in the open part of the model domain. A detailed description of the hydrodynamic model is found in Karlsson et al. (2010) and Eriksson and Engqvist (2013). The ecosystem and radionuclide food-web models for the present-day situation were implemented in MIKE using the ECO Lab software (DHI 2011), based mainly on in situ data collected during a single year (2004). Conceptually, the ecosystem model and the radionuclide transport model have been developed based on the general food-web structure introduced in earlier modeling studies within the area (e.g., Kumblad and Kautsky 2004), and for this article the high-resolution analysis considers only a limited number of selected radionuclides (Box S1, Electronic Supplementary Material).

### Source of Radionuclides

In the models, radionuclides are released into the environment from a point source (K-model) or number of distributed sources (D-model). The release of radionuclides is set at a constant rate of 1 Bq of each radionuclide per year. In the D-model, the sources were distributed according to the results of the groundwater modeling described in Berglund et al. (2013) and are mainly situated in basins 116, 117, 118, 120, and 121 (locations are shown in Fig. 3 of Kautsky et al. 2013). The sum of all the sources equals 1 Bq of each radionuclide per year. In the K-model, instantaneous homogeneous mixing of the radionuclides in the entire water volume is assumed. Hence, the point source in the K-model is included in the water phase directly, whereas the various sources in the D-model release the radionuclide to the sediment pore water from where it moves through the sediment to the water phase. The process of radioactive decay is not included in this study because of its minor importance for the environmental fate of these long-lived radionuclides. However, in the assessment model, decay and important decay chains are handled (Avila et al. 2013).

### Detailed Model Approaches

The two models share several features, such as the identity of compartments and state variables of the ecological model structure (Table 1), but the models differ greatly in how the underlying ecological models are executed. The structure and rates of the K-model are built on site-specific measurements of the biomasses of various functional groups and key species (see Kumblad et al. 2003, 2006) and corresponding rates of ecological processes, whereas the D-model is an ecosystem model (DHI 2011) that is adaptable to any aquatic ecosystem and has been used in numerous studies, and, for this study, was supplemented with four additional functional groups: planktivorous fish, deposit feeders, herbivores, and benthic predators.

**Table 1:** Characteristics of ecosystem K- and D-models used to simulate distribution of radionuclides in the Forsmark area, including basin 116. The compartment names in the K-model are indicated by bold type. Processes are indicated by italics. *DIC* Dissolved inorganic carbon, *PM* particulate matter, *POM* particulate organic matter, *DOM* dissolved organic matter

Model’s characteristics	K-model	D-model
Spatial resolution (basin)	1D model but allows adjacent 1D models (basins) to connected in a grid which gives a 2D representation	3D model: 180 horizontal boxes, 10 layers
Temporal resolution	Parameters integrated over 1 year; simulation time 100 years	3-h time step; 8 years simulation to reach quasi-stationary conditions
Physical exchange	Net in- and efflux across boundaries; hydrodynamics included as water turnover for the modeled basin	Fully dynamic driven by calibrated hydrodynamic model
Ecosystem model	8 State variables (shown below in bold)	17 Pelagic state variables and 26 benthic state variables
Inorganic solutes	**DIC**. A separate nutrient model calibrates primary production to nutrient accessibility	Carbon (DIC), nitrogen (***NO_2-3_ and NH_4_), phosphorous (PO_4_)
Primary producers	**Phytoplankton** (pelagic microalgae, pelagic heterotrophic bacteria, photosynthesising bacteria, cyanobacteria, diatoms, and dinoflagellates) **Benthophytes** (benthic microalgae, benthic macroalgae, phanerogams, bryophytes)	Pelagic microalgae Benthic microalgae Benthic macroalgae Phanerogams (benthic) Bryophytes (benthic)
Pelagic consumers and decomposers and *processes*	**Zooplankton** (Planktonic animals) **Fish** (demersal and pelagic) *Decomposition of detritus by pelagic heterotrophic bacteria is included in the phytoplankton compartment*	Zooplankton (grazers on phytoplankton) Fish (planktivorous; e.g., sprat) *Degradation of detritus (bacteria)*
Detritus	**PM** (pelagic and benthic)	POM/DOM (pelagic)
Benthic consumers and *processes*	**Grazers** (crustaceans and gastropods) on benthic macroalgae **Benthos** (Benthic filter-feeders: mussels, cockles, and clams; soft bottom macrofauna, i.e., deposit feeders and predators; meiofauna; benthic bacteria (*decomposers of organic matter*))	Grazers (crustaceans and gastropods) on benthic micro- and macroalgae *Benthic filter*-*feeding on phytoplankton* Deposit feeders (infauna in soft bottom) Benthic predators (e.g., *Saduria* and flounder) *Degradation of organic matter on seabed and in sediments*
Sediment	*Burial of radionuclides in sediment*	*Nutrient transformations* *Oxygen and redox dynamics* *Resuspension*–*sedimentation*

**Fig. 2:**
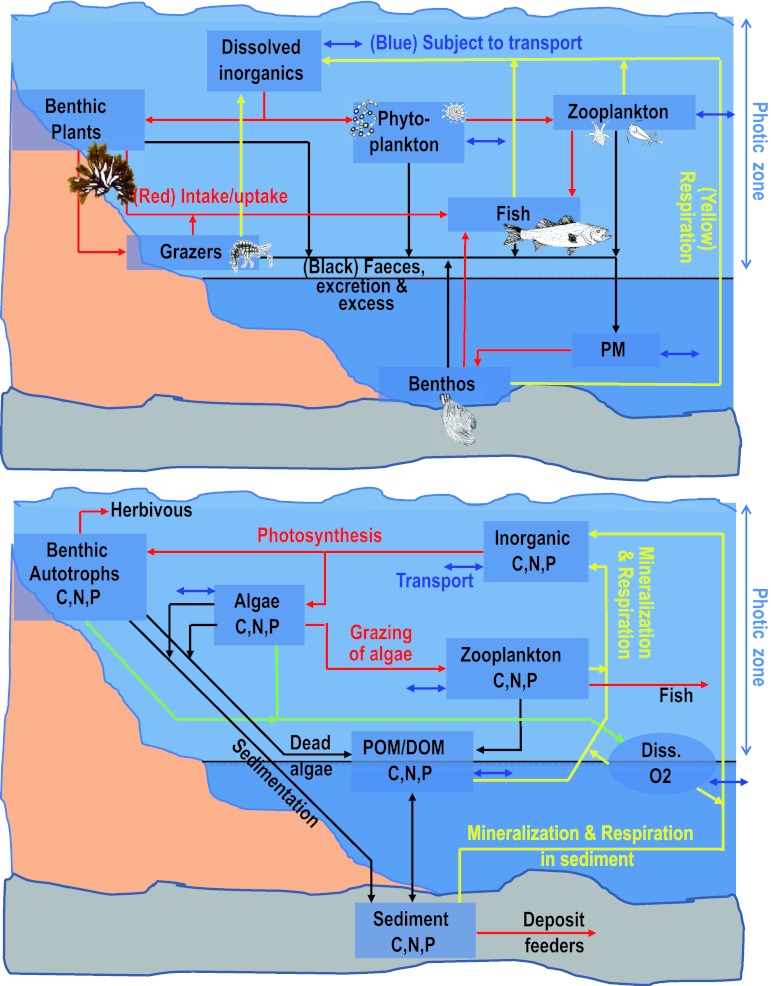
Schematic (simplified) structure of the ecosystem models used in the study; *upper panel* compartment model (K); *lower panel* 3-dimensional dynamic model (D). Note that only parts of the D-model are outlined in this figure. The details of the autotrophic model, sediment model including benthic filter-feeders, epibenthic grazers, deposit feeders, and predators as well as fish are not included. See Table 1 and Erichsen et al. (2010) for details. *PM* Particulate matter, *DOM* dissolved organic matter, *POM* particulate organic matter

Figure 2 shows schematic (simplified) structures of the two ecosystem models. The K-model operates with space-averaged biomasses and rates taking into account depth-dependent variations in the photic zone, and uses parameters (e.g., insolation, temperature) integrated over 1 year. Hence, the K-model maintains constant biomasses through simulation years. The K-model groups species having the same ecological functions into one biological compartment, thus reducing the number of state variables. The K-model is not calibrated, but the primary production is adjusted to a separately run-coupled nutrient model (Kumblad and Kautsky 2004) that takes into account the nutrients and water exchange across the boundaries (Eriksson and Engqvist 2013).

The two models share most of the same state variables or groups of state variables (Table 1), with the following exceptions: In the D-model, the effect of benthic filter-feeders was imposed by a spatially varying filtration of phytoplankton and particulate organic matter in the near-bed model layer according to the measured distribution of mussels, cockles, and clams. The effect of heterotrophic bacteria and meiofauna are implemented in the D-model as mineralization of carbon and nutrients, driven by the concentration and supply of particulate organic matter (POM) and temperature. Overall, the D-model is driven by light availability (insolation, light attenuation—including the effect of resuspension), nutrient availability (run-off, atmospheric deposition, sediment, and water column mineralization), and exchange across model boundaries (wind- and water-level-driven circulation). The D-model was calibrated against measurements of nutrients, chlorophyll, Secchi depth, and biomasses of benthic vegetation and deposit feeders (Erichsen et al. 2010).

In both models, radionuclides are assumed to follow the flow of organic carbon in the food web, and radionuclide relocation is regulated by several radionuclide-specific mechanisms: uptake by phytoplankton and benthic vegetation, adsorption to organic surfaces, and assimilation and excretion by animals. In contrast to the K-model, the D-model considers adsorption of radionuclides to suspended matter and sediments with different partition coefficients, *K*_d_s, for organic and inorganic particles. The D-model includes a sediment module consisting of two compartments with an upper active layer subject to re-suspension and a lower layer consisting of consolidated sediments. In sediments, radionuclides are adsorbed to organic and inorganic matters, dissolved in pore water and, depending on (modeled) oxygen penetration and redox conditions, certain radionuclides may precipitate (as sulfides or carbonates) or dissolve.

In both models, radionuclide-specific dynamics depend on partition coefficients, *K*_d_s, between radionuclides adsorbed to suspended particulate matter or organisms and in the surrounding water (see Box S1, Electronic Supplementary Material). As adsorption is directly proportional to the surface area, organisms with a high surface-to-volume ratio (*S*:*V*), such as phytoplankton, show high partition coefficients for adsorbing radionuclides. The *S*:*V* ratio for the different trophic state variables was calculated from the physical dimensions of dominant species within each trophic group assuming spherical, cylindrical, or flat geometrical forms according to their morphology. For fish, the total area of gills was additionally used to represent the adsorbing area (Pauly 1981). Total surface area within trophic groups was calculated from *S*:*V* ratio, biomass, and abundance based on monitoring data from Aquilonius (2010).

In both the K- and D-models, accumulated radionuclides are retained, and release takes place only when organisms die or are consumed. In the K-model, radionuclides have an additional efflux from organisms along with excreted feces.

Similarities and differences between the models are evaluated in this article by comparing modeled CRs, defined as the ratio of the radionuclide concentration in the organism resulting from all exposure pathways (including water, sediment, and dietary pathways) to the concentration in sea water (or pore water for infauna), normalized to the carbon content of the biota fraction and expressed as [(Bq kg^−1^ fw)/(kg_C_ per kg fw)] per (Bq m^−3^), i.e., kg_C _m^−3^, where fw is fresh weight. The CR definition assumes that the radionuclides in the organisms are in equilibrium with the ambient sea water. A large number of radionuclides that could hypothetically be released from the repository of radioactive waste have been modeled (Kumblad et al. 2006; Avila et al. 2010, 2013; Erichsen et al. 2010), but for this article, we have selected three radionuclides for comparison of model results: ^135^Cs, ^59^Ni, and ^230^Th. All three are long-lived radionuclides relevant to the planned high-level radioactive waste repository. Cs is also of interest due to its post-Chernobyl abundance as ^137^Cs in the Baltic Sea (HELCOM 2009). The element Ni is also biologically interesting as it is essential for many phytoplankton species (Muyssen et al. 2004). ^230^Th can be a naturally occurring daughter of ^238^U decay which is highly particle reactive, i.e., high *K*_d_, and does not assimilate in organic tissues. In contrast, Cs and Ni have around ten times lower *K*_d_ values than Th.

## Results and Discussion

### Spatial and Temporal Variations

**Fig. 3:**
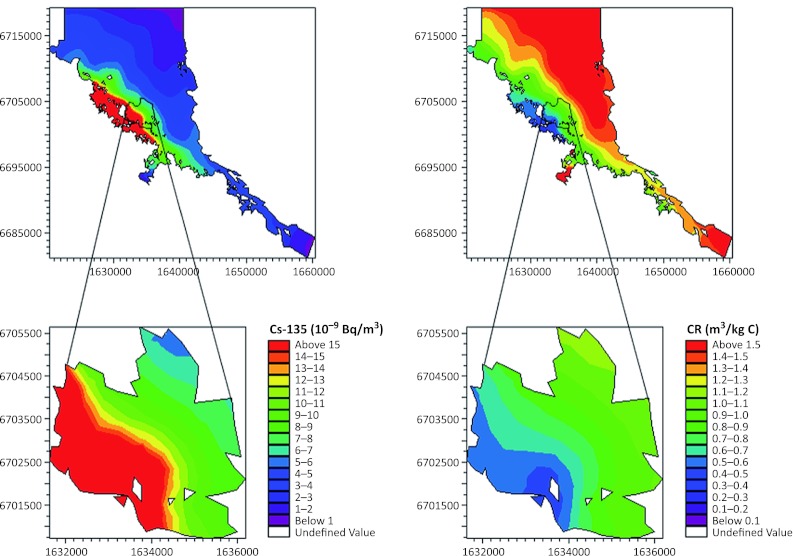
Modeled yearly average concentration of dissolved Cs-135 in water (10^−9^ Bq m^−3^) in the Forsmark area (*upper left*), basin 116 near the simulated source of 1 Bq y^−1^ (*lower left*), yearly average concentration ratio (CR) for phytoplankton (m^3^ kg C^−1^) in the Forsmark area (*upper right*), and basin 116 near the simulated outlet (*lower right*). Arrow (*lower right*) indicates position where time-series of CR values depicted in Fig. 4 was extracted

The D-model showed gradients in radionuclide concentrations in water and of CR in phytoplankton (Fig. 3), from the repository outlet to the open boundary of the Forsmark area. For example, within the Forsmark area the yearly averaged CR for Cs-135 in phytoplankton varied by a factor of 10–20, and this variation is explained primarily by the spatial variation in dissolved ^135^Cs concentration (Fig. 3). Even at smaller spatial scales (e.g., within basin 116) the D-model shows a threefold variation in yearly averaged CR for phytoplankton (Fig. 3).

**Fig. 4:**
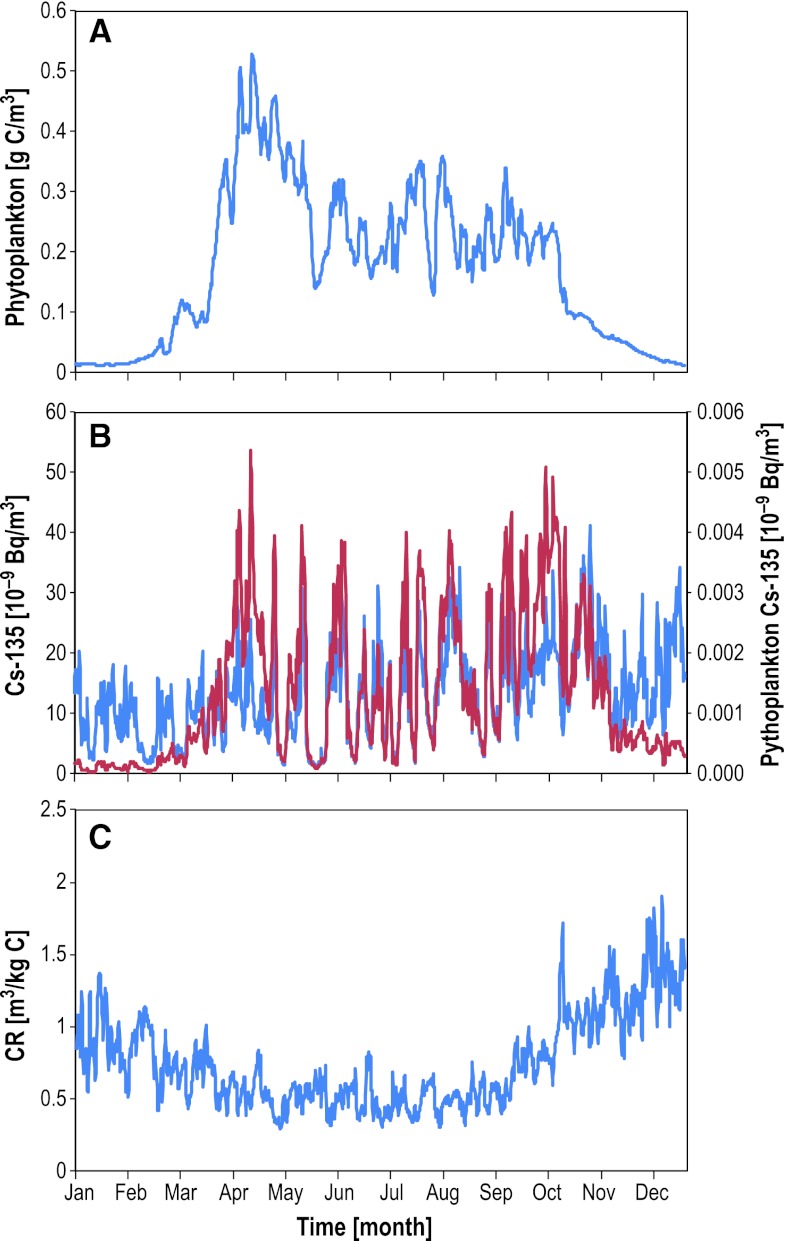
Modeled seasonal variation in phytoplankton biomass (**A**), ^135^Cs concentration in water (*blue*) and phytoplankton (*red*) (**B**), and concentration ratio for ^135^Cs in phytoplankton CR_ph_ (**C**). Data were extracted from the position shown in Fig. 3

In addition to spatial variation, the radionuclide concentration and CR in phytoplankton vary on a weekly timescale driven by water exchange, resulting in alternating conditions with water and plankton containing high concentrations (i.e., associated with the radionuclide plume) followed by low concentrations when uncontaminated water and plankton are passing the fixed site. Whereas the modeled phytoplankton biomass has a distinct seasonal variation (Fig. 4A), the seasonal variation in CR is somehow smaller with about two times higher CRs during winter than during summer (Fig. 4C), suggesting a negative influence of phytoplankton biomass on CR. Some of the variation in CR is explained by a varying turnover of phytoplankton biomass and the “slow” adsorption–desorption processes resulting in a loose coupling between changes in radionuclide concentrations in water and phytoplankton. Partial decoupling of ^135^Cs concentration in water and phytoplankton is especially visible during the spring bloom in April (Fig. 4B). The same patterns as shown in Fig. 4, though with different CRs, are found for the other radionuclides and other sites within the study area. Mass balance modeling and empirical measurements in the area also show this imbalance for many elements during the spring bloom (Bradshaw et al. 2012).

It is thus clear that the CR approach has major pitfalls when working with releases from point sources: (i) organisms are diluted with unpolluted ones and (ii) spatial and temporal distributions are not homogeneous in the case of a point source. This is not usually taken into account in risk assessment because measured CRs are for elements which are homogeneously distributed in the environment, i.e., there is no dilution by uncontaminated organisms, or they are measured in the laboratory, disregarding all these processes.

### Comparison of Modeled CR Values with Site Measurements

**Fig. 5:**
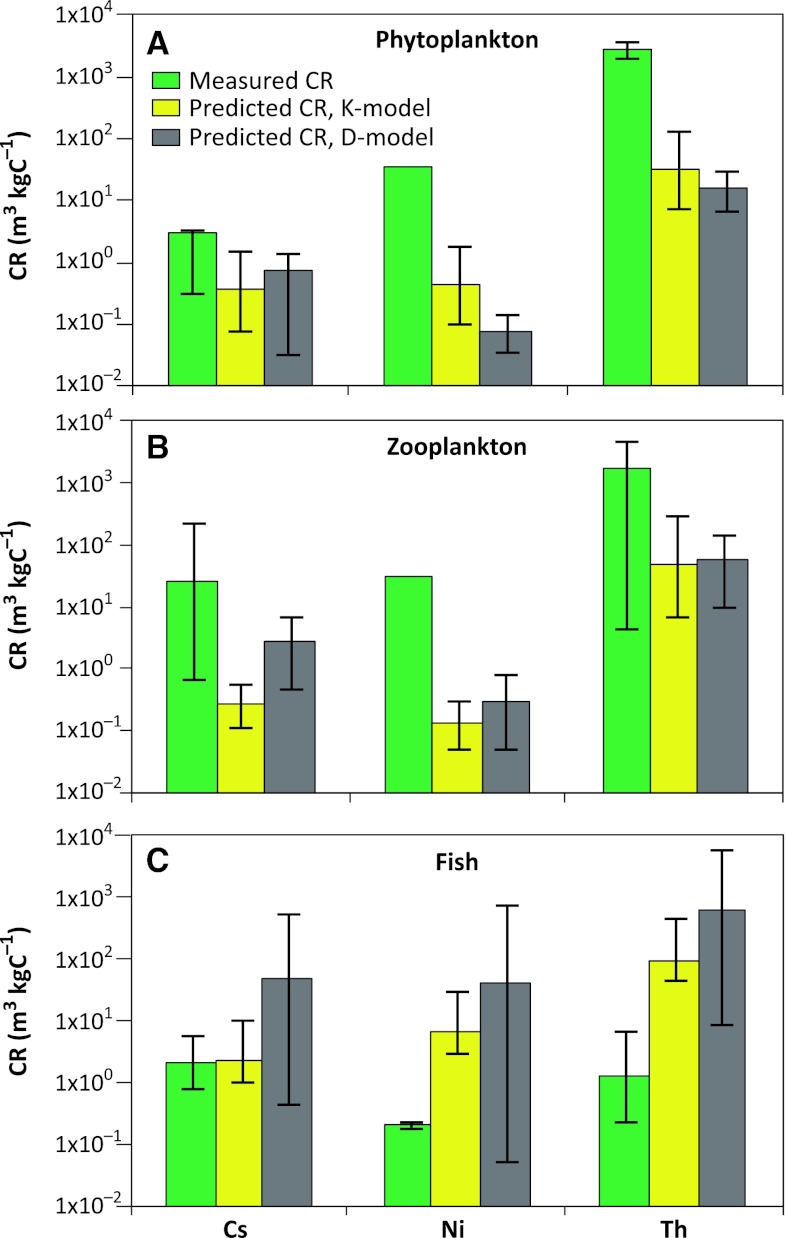
Comparison of 90 % CI and GM derived from measured data and modeled CR values of Cs, Ni, and Th for marine phytoplankton, zooplankton, and fish, respectively. K-model CRs are 50 % median with 5 and 95 % percentiles; D-model CRs are GM with 5 and 95 % percentiles. The D-model has both temporal and spatial percentile intervals. In the figure, the largest percentile intervals are included, hence, the temporal variation is shown for phytoplankton and zooplankton, but spatial variation is shown for fish

The results of the two model simulations of the CR of phytoplankton, zooplankton, and fish for the three elements ^135^Cs, ^59^Ni, and ^230^Th are presented in Table 2 and in Fig. 5, where they are compared with site-specific CR values. Overall, the predicted results from models K and D were in reasonable agreement considering the differences in applied distribution constants and model structures.

**Table 2:** Statistics including GM geometric mean (GM) or median (50 % percentile) and confidence interval (95 % CI) of concentration ratios CR (m^3^ kg C^−1^) for ^59^Ni, ^135^Cs, and ^230^Th in phytoplankton, zooplankton, and fish, as estimated in model simulations (K-model and D-model) and measured in the Forsmark area. The measured CR values are from Nordén et al. (2010) and those marked * are from Kumblad and Bradshaw (2008)

Isotope	CR measurements			CR predicted by K-model			CR predicted by D-model				
	GM	95 % CI		Median 50 %	95 % CI		GM	Spatial 95 % CI		Temporal 95 % CI	
		Lower	Upper		Lower	Upper		Lower	Upper	Lower	Upper
Phytoplankton											
Ni-59	3.70E+01*	–	–	4.44E−01	1.02E−01	1.87E+00	7.77E−02	4.98E−02	1.17E−01	3.68E−02	1.52E−01
Cs-135	3.00E+00	3.30E−01	3.30E+00	3.58E−01	8.19E−02	1.50E+00	7.15E−01	4.54E−01	1.10E+00	3.37E−01	1.39E+00
Th-230	2.70E+03	2.00E+03	3.64E+03	3.23E+01	7.39E+00	1.36E+02	1.51E+01	9.63E+00	2.29E+01	7.01E+00	2.98E+01
Zooplankton											
Ni-59	3.10E+01*	–	–	1.30E−01	5.34E−02	3.06E−01	3.06E−01	1.70E−01	5.37E−01	5.21E−02	8.01E−01
Cs-135	2.56E+01	6.98E−01	2.30E+02	2.73E−01	1.18E−01	6.00E−01	2.66E+00	1.44E+00	4.86E+00	4.75E−01	6.75E+00
Th-230	3.20E+01	4.65E+00	4.65E+03	4.60E+01	7.25E+00	2.88E+02	5.93E+01	3.23E+01	1.02E+02	1.03E+01	1.51E+02
Fish											
Ni-59	2.10E−01*	1.90E−01	2.50E−01	7.01E+00	3.14E+00	3.20E+01	4.20E+01	5.78E−02	7.43E+02	9.85E+00	1.31E+02
Cs-135	2.20E+00	8.30E−01	5.80E+00	2.32E+00	1.09E+00	1.08E+01	4.75E+01	4.87E−01	5.53E+02	1.12E+01	1.49E+02
Th-230	1.30E+00	2.50E−01	6.90E+00	9.81E+01	4.56E+01	4.67E+02	6.21E+02	9.35E+00	5.92E+03	1.40E+02	1.94E+03

#### Cesium

The modeled CR values from the K- and D-models for Cs were in good agreement with experimental site-specific data (Table 2) for all three groups of organisms (Fig. 5). Only the CR value for zooplankton estimated by the K-model was close to the lower limit of measured CR values. Some studies have suggested biomagnification of radiocesium in both marine (Kasamatsu and Ishikawa 1997) and freshwater (Rowan and Rasmussen 1994; Smith et al. 2003) food webs, but there was no evidence for this in this study (Table 2) and previous modeling by Kumblad et al. (2006) and Bradshaw et al. (2012) has shown that it is probably of minor importance.

#### Nickel

Both models underestimated CR values for phyto- and zooplankton by one to two orders of magnitude. This may be because Ni is a biologically essential element for cyanobacteria and phytoplankton (Muyssen et al. 2004). Specific physiological regulation mechanisms for Ni uptake may thus be involved, which are outside the scope of the K- and D-models. Modeled CR values for Ni in fish were generally high and here biological processes may also be involved; fish may actively regulate its uptake and elimination (Phillips and Rainbow 1989; Muyssen et al. 2004). Assimilation and elimination are not accounted for in the D-model and only assimilation efficiency is included in the K-model, which may be the explanation of these overestimates.

#### Thorium

Modeled CR values of Th for zooplankton were in a good agreement with measured site-specific data (Fig. 5). As Th is particle reactive but not assimilated in organisms, uptake of Th by zooplankton is a function of passive adsorption onto the organisms’ surface (Rodriguez y Baena et al. 2007; Stewart et al. 2008) and thus predicted well by these models. It is, therefore, surprising that the modeled CR values of Th for phytoplankton were lower than measured values by one to two orders of magnitude. The explanation is probably that the site values are too high; few measurements were available and concentrations were often near the limit of detection for water concentrations, adding considerable uncertainty. Smaller organisms with higher *S*:*V* ratios typically display higher Th CR values than larger organisms (Stewart et al. 2008); this holds true for the measured zooplankton and fish data. Modeled Th CR values for fish are overestimated, but the lower modeled limit is in the same range as maximum measured values.

For all radionuclides, but especially those present at close to detection limits, it is difficult to obtain sufficient site measurements for phyto- and zooplankton. Large sample amounts are required for the analyses, and this may require much time and effort in the field. However, more field data are essential to improve the estimation and validation of CRs in these organisms.

### 3D Dynamic Model (D-Model) Versus Compartment Model (K-Model)

Despite differences in the model structures and input constants, modeled CR values for the three organism groups and three elements presented in this article were in reasonable agreement, suggesting that both models are robust. However, the two different models have strengths and weaknesses regarding realism and practicability that make them more or less applicable to different needs. The D-model allows estimates at a high spatial and temporal resolution (down to 20 m and over a few hours). This gives insights in how potential hotspots of exposure in space and time (e.g., algal blooms and localized radionuclide releases) can be identified or modeled, and thus a better assessment made of risks to organisms in the ecosystem and nearby areas.

However, the complex numerical calculations cause long computational times and thus limit the simulation period to decades, not centuries. Such precision may not always be necessary in a risk assessment, but is useful when specific events, measurements, or local heterogeneity need to be more accurately considered and assessed on shorter time scales (Harms et al. 2003). The high resolution gives an estimate of how large the errors are with coarser resolutions and helps to explore how, e.g., CR varies over space and time. The D-model is constrained by the mass balance of nutrients and organic matter, which means that estimated ecosystems are scaled properly to carbon- and water turnover.

Compartment models such as the K-model do not include hydrodynamics explicitly, but are based on the assumption that average water retention time from hydrodynamic models and/or field data are sufficiently good approximations for estimating the annual dynamics of the ecosystem. The K-model also assumes instantaneous and homogeneous distribution (over a year) of the radionuclides released within the compartment. This is not the case in reality, but seems to be of minor importance, varying the CR within the compartment within a factor of three. Variation of parameters in space and time is taken into account by consideration of their probability distribution functions, and applying the probabilistic simulations in the box model is much quicker and easier than in the 3D model. This also enables the K-model to make estimates over a longer time period (i.e., >100 years), which is necessary for the risk assessment of long-lived radionuclides. The structure of the K-model is such that it can be run at any spatial or temporal scale comparable to that of the D-model.

However, it requires input parameters for water exchange scaled to the same area that requires a water exchange model which is similar to MIKE-3, as used for the D-model. The mass balance of organic matter in the current K-model needs to be scaled by nutrient mass balance from a separate model (Kumblad and Kautsky 2004). Thus, the K-model requires more effort to manually scale the input parameters to proper temporal and spatial contexts.

A strength of both models is that they allow the incorporation of many biological compartments, food-web processes, and specific interactions and transfers such as feeding, respiration, death, excretion, etc., adding to the ecological realism of the model. In addition, they allow the calculation of the range and confidence limits of output values such as CR values, instead of simply producing mean values. This both allows the degree of confidence in the values to be assessed and also enables the identification of potentially high values that may lead to unwanted exposures.

The two models have been developed separately, but could be used to complement each other in different ways:

1. the K-model could provide results decades ahead, producing D-model input maps (initial values/fields) for a future situation that could then be analyzed in detail with the D-model;
2. the D-model, with its greater spatial and temporal resolutions, could identify areas of concern that could then be modeled on a longer time scale with the K-model;
3. probability density functions, PDFs, for *K*_d_s could be included in the 3D approach, which could then provide PDFs that included accumulated variations in *K*_d_ in time and space;
4. the D-model can serve as a partly independent validation of the K-model.

The mechanistic approach used in the models also shows that the need for element-specific data for many organisms can be reduced to favor more site-specific, high quality, and well-controlled measurements of a few organisms. The D-model also shows that the traditional approach with CR models has several flaws regarding the nature of point sources, and shows that CR measurements should be treated carefully with respect to temporal and spatial representations. Both models are based on substantial amounts of field data from the Forsmark site. The D-model uses flow fields based on real topography of the Forsmark area and realistic forcing functions such as wind and density, and both models use field data on organism biomass distributions, insolation, etc. This undoubtedly contributes to their robustness, but also requires substantial underlying scientific and financial input. This is not always possible in all risk assessment contexts, but in large-scale investigations such as those required prior to the building of a deep geological repository, much of this data must be collected. Thus, by proper planning and project management any extra data required for such modeling can be obtained at marginal extra effort and cost.

## Conclusions

- The CR concept is difficult to apply to releases from a point source, as radionuclide distributions are highly heterogeneous in space and time and contaminated organisms are mixed with uncontaminated individuals. Thus, if an accident or point source of radionuclides is assessed, measurements of CR are very uncertain.
- Both models calculate the range and/or confidence limits of CR values, an improvement over models that only estimate means.
- Both models include many ecological compartments and processes, adding to the ecological realism of the models and their output.
- The D-model is dynamic and 3D and enables high-resolution estimates of concentrations and CRs in space and time, allowing estimates of the heterogeneity of radionuclide distributions in the ecosystem and nearby areas. However, it is computationally heavy, making long-term modeling difficult. For assessment of point sources or accidents and short-term assessment, the use of a dynamic modeling approach provides valuable data.
- The K-model includes hydrodynamics as water turnover for the whole modeled area, includes realistic ecological parameters, and takes spatial and temporal variations into account by calculating probability distribution functions. It is computationally faster, allowing estimates over a period of >100 years, which are important when considering long-lived radionuclides. For assessment of uniformly distributed concentrations of biomass and radionuclides, and for long-term assessments, a compartment model is useful.
- The two model approaches could be combined in future studies to make use of their complementary strengths (long-term estimates from the K-model and high spatial and temporal resolutions of the D-model).

## Electronic Supplementary Material

Below is the link to the electronic supplementary material.

Supplementary material 1 (PDF 21 kb)
