# Supplementary material for: Radionuclide Transport and Uptake in Coastal Aquatic Ecosystems: A Comparison of a 3D Dynamic Model and a Compartment Model
Source: Ambio. 2013 Apr 26;42(4):464–75. doi: 10.1007/s13280-013-0398-2 (PMC3636370; doi:10.1007/s13280-013-0398-2)
Supplement: Supplementary file 1 — Supplementary material 1 (PDF 21 kb) [file 13280_2013_398_MOESM1_ESM.pdf]

Electronic Supplementary Material

Radionuclide Transport and Uptake in Coastal Aquatic Ecosystems – a Comparison of a 3D Dynamic Model and a Compartment Model

Anders Christian Erichsen, Lena Konovalenko, Flemming Møhlenberg, Rikke Margrethe Closter, Clare Bradshaw, Karin Aquilonius, Ulrik Kautsky

BOX S1: PARTITION COEFFICIENTS (K_d)

In safety assessment of nuclear facilities, solid/liquid partition coefficients (K_d) have been widely used to describe the sorption and retention of radionuclides (Sibley and Myttenere, 1986). In this paper, the K-model uses a single K_d value for total suspended particulate matter (PM), whereas the D-model uses separate K_d values for organic carbon ($K_{d\ C}$) and inorganic matter such as clay ($K_{d\ in}$).

In the **K-model,** K_d is defined as the ratio between the element concentration in suspended particulate matter C_p and the concentration in water C_w, i.e.,

$$K_d = C_p / C_w$$

and is expressed as (Bq kg dw^{-1}) per (Bq m^{-3}), i.e., m^3 kg dw^{-1} where dw is dry weight. The partition coefficient for particulate organic carbon (POC), $K_{d\ POC}$ ((Bq g$_C^{-1}$) per (Bq m^{-3})) is the product of the K_d (m^3 kg dw^{-1}) and a conversion factor which is the ratio of the mass (kg dw) of suspended particulate matter to its carbon content (g): 0.006 (kg dw g$_C^{-1}$). The K_ds used in the K-model are site-specific (Nordén et al. 2010) as shown in Table S1.

The **D-model** uses separate coefficients for organic carbon ($K_{d\ C}$) and inorganic material ($K_{d\ in}$). $K_{d\ C}$ and $K_{d\ in}$ were estimated from literature values of relations between element concentrations in 'muddy' sediments and their organic carbon content, where the intercept (zero organic carbon) is used to estimate $K_{d\ in}$ and the slope is used to estimate $K_{d\ C}$. Being dynamic, the D-model resolves adsorption-desorption processes explicitly in each model cell, taking account of summed surface areas for inorganic and organic matter and imports and exports of radionuclides across grid cell boundaries, and uses K_d as an equilibrium representation of the ratio between adsorption rate and desorption rate. Modeled rates of adsorption and desorption of radionuclides are controlled at low concentrations using a Michaelis-Menten relation to fulfill Courant–Friedrichs–Lewy conditions (Erichsen et al. 2010).

Table S1 Model constants for partition coefficients (K_d) in model D and K. Note that units differ between the two models. The K-model follows the carbon cycle estimated as dry weight, whereas the D-model follows the carbon cycle estimated as both C and inorganic concentrations

**Radionuclide**	**D-model**		**K-model**
	**$K_{d\ C}$** **m^3 kg C^{-1}**	**$K_{d\ in}$** **m^3 kg dw^{-1}**	**K_d** **m^3 kg dw^{-1}**
**Cs-135**	20	0.13	19
**Ni-59**	2.1	0.015	14
**Th-230**	417	1.67	740

REFERENCES

Erichsen, A.C., F. Møhlenberg, R.M. Closter, and J. Sandberg. 2010. Models for transport and fate of carbon, nutrients and radionuclides in the aquatic ecosystem at Öregrundsgrepen. Svensk Kärnbränslehantering AB, SKB R-10-10, Stockholm, Sweden, Report, 95 pp.

Nordén, S., R. Avila, I. de la Cruz, K. Stenberg, and S. Grolander. 2010. Element specific and constants parameters used for dose calculations in SR-Site. Svensk Kärnbränslehantering AB, SKB TR-10-07, Stockholm, Sweden, Report, 123 pp.

Sibley, T. H., and C. Myttenere, eds. 1986. *Application of distribution coefficients to radiological assessment models.* Oxford: Elsevier Science Ltd., 430 pp
